# Valedictory

**Published:** 1888-07

**Authors:** W. C. Barrett


### Editorial.

### VALEDICTORY.

With this number The Independent Practitioner opens a new chapter in its not uneventful history. Like a fair bride, it leaves the home which it has known so long, and the care of those who have tenderly watched its budding growth, and becomes the center of another circle, the object of the affections of a new relationship. The simile seems not inapt, for the journal has passed the period of its immaturity. It was born in southern climes, and its days of infancy were passed in scenes unfamiliar to its later years. It was not a particularly healthy or promising child, but it was always bright and interesting. When it came under the care of its late guardians, and was removed to the more bracing, if not so genial, atmosphere of its northern home, it began straightway to grow at an encouraging rate, and has developed into a strong and ruddy life. Its days of childhood passed, and those of full adult life having been reached, it no longer can rest content with being but an inmate, albeit a loved one, of another's home, dividing the care and attention with other and older children, but demands a separate establishment and the exclusive devotion of him to whom it is entrusted. It may even be that it shall take upon itself another name—that of its new-made relations. But though it may go out from its old home, it will not cease to be the object of the love and solicitude of its old sponsors, but has rather only enlarged its circle of friends and drawn about it a yet wider household.

To abandon the figure and come down to plain facts, the Independent Practitioner has outgrown its surroundings. Its readers will bear witness that it has not been given to undue boastfulness in the past, and it may therefore be permitted a few appreciative words at the present.

When the editor, six and one-half years ago, first became connected with it, the Journal was published by its founder, Dr. B. M. Wilkerson, and it had both medical and dental departments. But two or three numbers were issued when the medical editor resigned, and the dental editor assumed the sole direction of its pages. About the same time Dr. Wilkerson found himself obliged to withdraw from the undertaking, and the Journal passed into the hands of its editor, who, however, soon found himself unable to carry the burthen alone. A syndicate of dentists was therefore formed, and with a very few changes this Association has since conducted the journal in entire unanimity of purpose and in unbroken harmony. Commencing with a mere handful of paying subscribers, they have seen the almost unknown journal grow into an established and honored position, until to-day it stands at the head of independent journalism, with a name that is known and respected wherever educated dentistry has an existence. It has always labored for the good of the profession which it represented, and has always kept its record clean. With each half year it became necessary to increase the size of the edition, and its business relations were constantly augmented, until now few realize the magnitude of the labor connected with its issue.

All of those who have been actively connected with it are dentists in full practice, and the only time that could be devoted to journalism were the moments stolen from sleep and honestly earned rest. The hour has come when the editor, especially, upon whom the main labor has fallen, must choose between a practice which demands all his attention, and the duties attendant upon his office. He is not competent for both, and has already made too large drafts upon his physical life. He chooses to stick to his earliest love, and give to dentistry all that there is in him, and therefore he retires from the editorial chair. The INDEPENDENT PRACTITIONER has been largely the child of his brain and his affection, and to attempt to convey the impression that he leaves it without regret would be mere affectation ; and he desires to carry with him the respect and regard of all who have so long patiently listened to him, and he hopes and trusts that there are many readers who will part from him with regret. That he has made mistakes no one knows better than he, for he had his trade to learn, but he knows they were errors of the head and not of the heart, for the best interests of dentistry have ever been his honest aim.

He cannot allow this opportunity to pass without rendering to his late associates the homage of a grateful heart for their constant and unwearied forbearance—too often sorely tried he fears—and for their unfailing support and sympathy when, during the years of the past, they were so urgently needed. He has tried always to be guided by their advice, but unfortunately they were not at his immediate elbow, and in consequence he was too often obliged to assume a responsibility and be answerable for results which their better judgment might have beneficially modified. For six years have we worked in harmony, without a misunderstanding to break the monotony, and the editor had really begun to believe that there must be something of good in him or he could not have secured the friendship of such men as composed the New York Dental Journal Association. Trusting that the journal has but entered upon a new career of prosperity, commending the new editor and his associates to his old readers and assuring them that his interest in the INDEPENDENT PRACTITIONER is still an active one, the old editor resigns the helm to one who proposes to give to the journal his undivided attention. W. C. BARRETT.

